# NURR1 Downregulation Favors Osteoblastic Differentiation of MSCs

**DOI:** 10.1155/2017/7617048

**Published:** 2017-07-09

**Authors:** Adriana Di Benedetto, Francesca Posa, Claudia Carbone, Stefania Cantore, Giacomina Brunetti, Matteo Centonze, Maria Grano, Lorenzo Lo Muzio, Elisabetta A. Cavalcanti-Adam, Giorgio Mori

**Affiliations:** ^1^Department of Clinical and Experimental Medicine, Medical School, University of Foggia, Foggia, Italy; ^2^Institute of Physical Chemistry, Department of Biophysical Chemistry, University of Heidelberg and Max Planck Institute for Intelligent Systems, Stuttgart, Germany; ^3^Department of Basic and Medical Sciences, Neurosciences and Sense Organs, University of Bari, Bari, Italy; ^4^IRCCS Istituto Tumori Giovannoi Paolo II, Bari, Italy; ^5^Department of Emergency and Organ Transplantation, University of Bari, Bari, Italy

## Abstract

Mesenchymal stem cells (MSCs) have been identified in human dental tissues. Dental pulp stem cells (DPSCs) were classified within MSC family, are multipotent, can be isolated from adult teeth, and have been shown to differentiate, under particular conditions, into various cell types including osteoblasts. In this work, we investigated how the differentiation process of DPSCs toward osteoblasts is controlled. Recent literature data attributed to the nuclear receptor related 1 (NURR1), a still unclarified role in osteoblast differentiation, while NURR1 is primarily involved in dopaminergic neuron differentiation and activity. Thus, in order to verify if NURR1 had a role in DPSC osteoblastic differentiation, we silenced it during all the processes and compared the expression of the main osteoblastic markers with control cultures. Our results showed that the inhibition of NURR1 significantly increased the expression of osteoblast markers collagen I and alkaline phosphatase. Further, in long time cultures, the mineral matrix deposition was strongly enhanced in NURR1-silenced cultures. These results suggest that NURR1 plays a key role in switching DPSC differentiation toward osteoblasts rather than neuronal or even other cell lines. In conclusion, DPSCs represent a source of osteoblast-like cells and downregulation of NURR1 strongly prompted their differentiation toward the osteoblastogenesis process.

## 1. Introduction

The regenerative medicine is increasing its interest in using adult stem cells for the regeneration of mineralized tissues. Specifically, wide variety of postnatal MSCs have been identified in the dental tissues in the past decade. In particular, DPSCs can be isolated from the dental pulp of adults, a tissue containing the progenitors of the dentinogenic lineage and thus physiologically involved in the reparative processes of dentin [1–3]. Although the regenerative process of the dentin/pulp complex is not well understood, it is known that the reparative dentin is deposed as a protective barrier for the pulp as a consequence of trauma or cavity [4, 5]. DPSCs are normally quiescent, but, following injuries that cause odontoblast death, they can resume their biological activity. Thus, in response to stimuli located on pulp-dentin interface, DPSCs are recruited at the site of the lesion and differentiate into odontoblasts synthesizing reparative dentin and preserving tooth vitality. Previous works showed that DPSCs can be considered odontoblast/osteoblast precursors because they express osteogenic markers and are responsive to many growth factors for osteo/odontogenic differentiation [6–8]. In addition, dental pulp cells are capable of forming mineral matrix nodules [2, 9–11]. Actually, it has been demonstrated that DPSCs can differentiate toward multiple cell lineages; hence, when stimulated with the appropriate culture media, they showed the capacity to differentiate into chondrocyte-like, adipocyte-like, and osteoblast-like cells [12–17]. Consistently, more studies showed that DPSCs, when properly stimulated, can be induced to differentiate into neuronal-like and glial cells expressing the typical markers nestin and glial fibrillary acidic protein (GFAP) [18–21]. In addition, DPSCs showed to differentiate into osteoblast-like cells, express the main bone matrix protein collagen I (Col1), the typical osteoblast enzyme alkaline phosphatase (ALP), and form nodules of mineralized matrix [2, 15, 22–24]. This suggests the presence of different niches of progenitors/stem cells in the pulp with a multipotency of differentiation that can be intercepted and altered by the appropriate stimuli. Morphological characteristics of DPSCs were compared to those of mesenchymal stem cells (MSCs) from bone marrow; the comparison showed many similarities [2, 13]; it is also relevant that gene expression profiles of the two cell populations were very similar [25–27]. The finding of the differentiation potential of DPSCs led the scientists to consider them as an alternative source of postnatal stem cells. In particular, the ability to differentiate into osteoblast-like cells, which are able to deposit a mineralized matrix, has revolutionized the dental research and opened new perspectives for reconstructive surgery and calcified tissue bioengineering. The literature data on dental stem cells are so promising that American companies, with the approval by the Food and Drug Administration (FDA), provide a service of isolation and preservation of these cells where the onset of disease would make their use beneficial in therapy. Although the plasticity of DPSCs and their ability to generate many different cell lines are already known, what genes are involved in the multilineage differentiation ability of these cells and in their osteoblastic differentiation process remains unclear and needs to be deeply investigated, since osteoblastogenesis is influenced by many cytokines and genes [28, 29]. We have reported in a previous work that DPSCs express the nuclear receptor NURR1 in basal and in osteogenic conditions [23], a surprising finding, considering that NURR1 is a member of the nuclear steroid/thyroid receptor superfamily, expressed primarily in the central nervous system, essential for the survival and development function of dopaminergic neurons of the ventral nuclei of the brain [30]. Indeed, the expression of NURR1 was already described in DPSCs and SHEDs, but a role for the receptor was mostly attributed during the differentiation toward a neuronal phenotype [19, 31–33]. Actually, a couple of works reported, in mice calvarial osteoblast and MC3T3-E1, that NURR1 increased the expression of osteoblastic markers [34, 35]. Conversely, a more recent work described a cross talk between NURR1 and *β*-catenin where NURR1 inhibited *β*-catenin-mediated expression and *β*-catenin was capable of inhibiting the transcriptional activity of NURR1 [36]. So far, NURR1 is expressed in DPSCs, but its role in the osteogenic differentiation is still controversial and needs more investigations. Thus, having established that DPSCs are an excellent model for studying the osteoblast differentiation [2, 15, 22–24], in this work, we knock down NURR1 in DPSCs, by using the gene silencing technology, and elucidated the effect and the role of Nurr1 in osteoblast differentiation.

## 2. Results

### 2.1. Osteogenic Trigger Inhibits Neuronal Markers Expression in DPSCs

**Figure 1:**
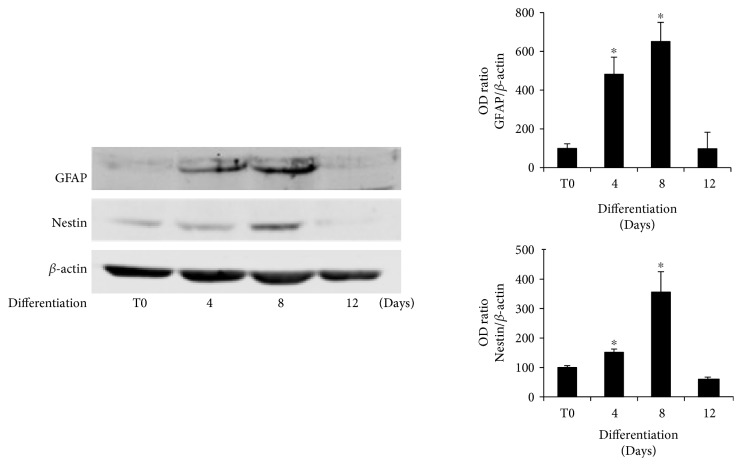
Expression of GFAP and nestin during osteogenic differentiation of DPSCs. Immunoblots show the protein expression trend of GFAP and nestin in DPSCs cultivated in osteogenic conditions for 4, 8, and 12 days. Both proteins were expressed during the first phases of osteogenic differentiation (4–8 days), but their expression dramatically dropped after 8 days of culture. Data are presented as means ± SE of 3 independent donors. ^∗^*P* < 0.01 compared to T0. Statistics: unpaired Student's *t*-test.

To confirm that DPSCs, following the osteogenic differentiation treatment, commit to osteoblastic lineage and lose their multipotency, we analyzed the expression of the neuronal protein nestin and the astrocytes marker GFAP. The cells were cultured in presence of osteogenic media, and the total cell lysates were collected at different time points (T0, 4, 8, and 12 days) to be analyzed by Western blotting.  Figure 1() shows that both nestin and GFAP are expressed during the first phases of osteogenic differentiation, but their expression became dramatically reduced after 8 days of culture. These results demonstrated that, during the first days (4–8) of osteogenic differentiation, DPSCs continue to maintain neural potentials, or perhaps not all the cells are already committed while, after 8 days of culture in osteogenic medium, the neuronal potential of DPSCs appeared completely suppressed.

### 2.2. NURR1 Expression Was Knocked Down in DPSCs

**Figure 2:**
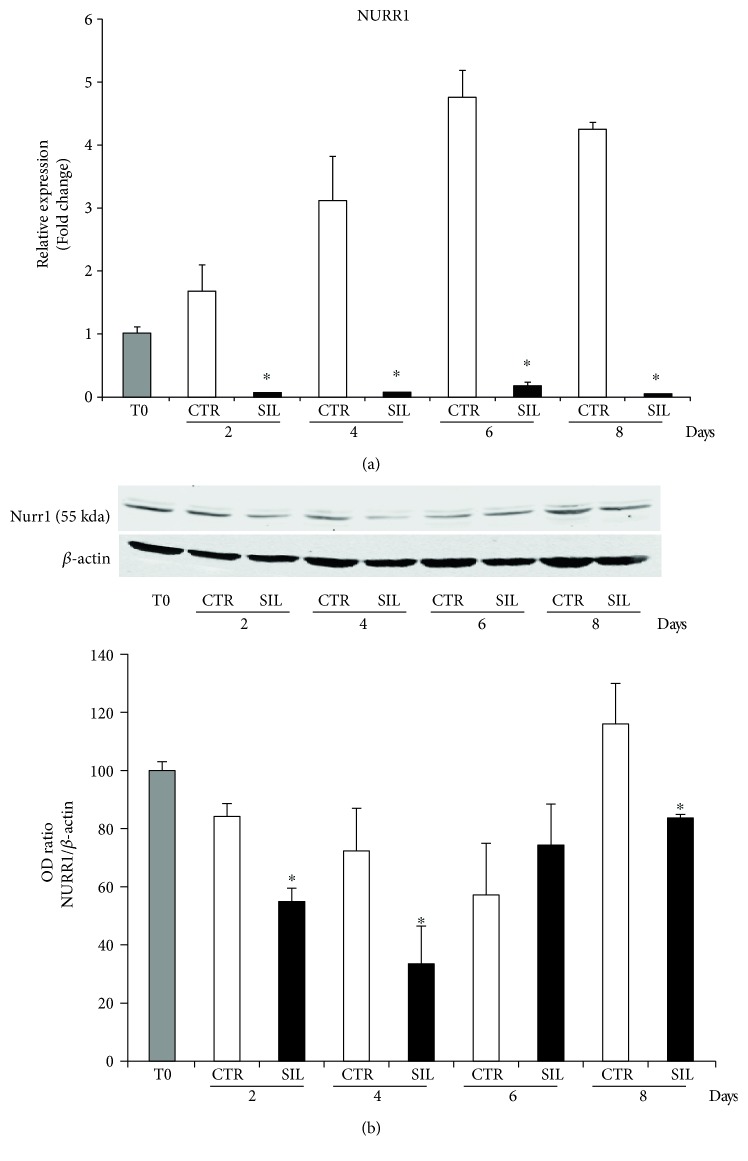
Effect of NURR1 short-interfering knockdown. (a) qPCR of DPSCs differentiated in osteogenic conditions and transfected with NURR1-specific siRNA (black bars) or scrambled sequences as control (white bars) showed the effective knockdown of NURR1 mRNA. A time course demonstrated that NURR1 expression remained downregulated along the culture. Expression was normalized to GAPDH. ^∗^*P* < 0.01 compared to CTR. (b) NURR1 mRNA downregulation was confirmed and validated by Western blotting indicating that NURR1 protein was knocked down. Each graph represents means ± SE of 3 independent donors. ^∗^*P* < 0.01 compared to CTR. Student's 𝑡-test was used for single comparison.

Our previous work, showing that NURR1 was expressed in DPSCs in basal conditions and still present when the cells differentiated into osteoblast-like cells [23], prompted us to deeper investigate the role of NURR1 in DPSCs during the differentiation toward osteoblastic lineage. To this purpose, we used siRNA to knock down NURR1 expression in DPSCs from time zero (T0) during the whole differentiation process. The cells were seeded in osteogenic medium and the silencing sequences NURR1 (SIL) or scramble (CTR) were added every 48 hrs in order to keep NURR1 downregulated. All cell lysates were collected and subjected to qPCR showing a dramatic reduction of Nurr1 mRNA in silenced samples relative to CTR at the all analyzed time points (2, 4, 6, and 8 days) (Figure 2(a)). Detection of NURR1 protein levels was performed by Western blotting, confirming the decrease of the protein in NURR1 silenced cells (Figure 2(b)).

### 2.3. NURR1 Downregulation Favors the Osteogenic Differentiation of DPSCs

Once verified that NURR1expression was silenced during the osteoblastic differentiation of DPSCs, we analyzed how NURR1 knockdown could influence the osteogenic differentiation of DPSCs. Osteoblastic markers such as ALP, Col1, Runx-2, osteoprotegerin (OPG), osteopontin (OPN), and osteocalcin (OCN) were studied by qPCR: a schematic panel of the results is shown in Table 1.

**Figure 3:**
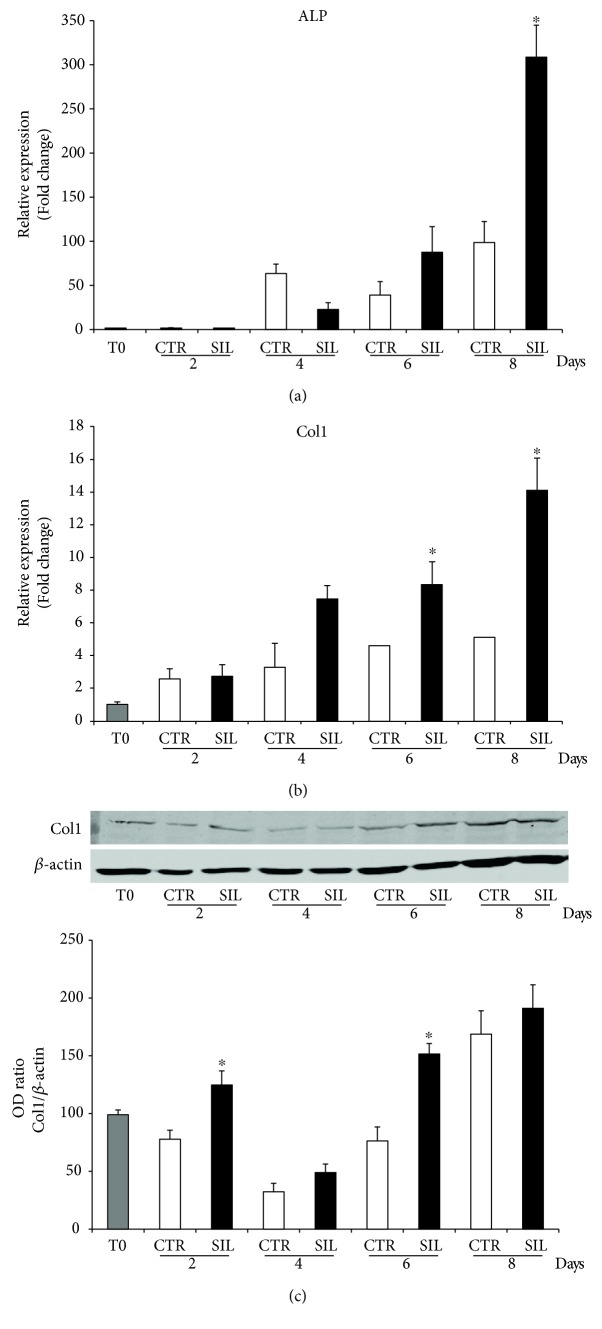
Effect of NURR1 downregulation on osteoblast markers. (a)-(b) qPCR performed on si-NURR1 or CTR cells showed that NURR1 downregulation significantly increased the expression of the two osteoblast markers ALP (8 days) (a) and Col1 (6–8 days) (b). Expression was normalized to GAPDH. ^∗^*P* < 0.01 compared to CTR. (c) Immunoblotting confirmed that the expression of Col1 protein increased in NURR1 silenced cells relative to CTR cells (^∗^*P* < 0.01). Each graph represents means ± SE of 3 independent donors. Statistics: unpaired Student's *t*-test.

**Figure 4:**
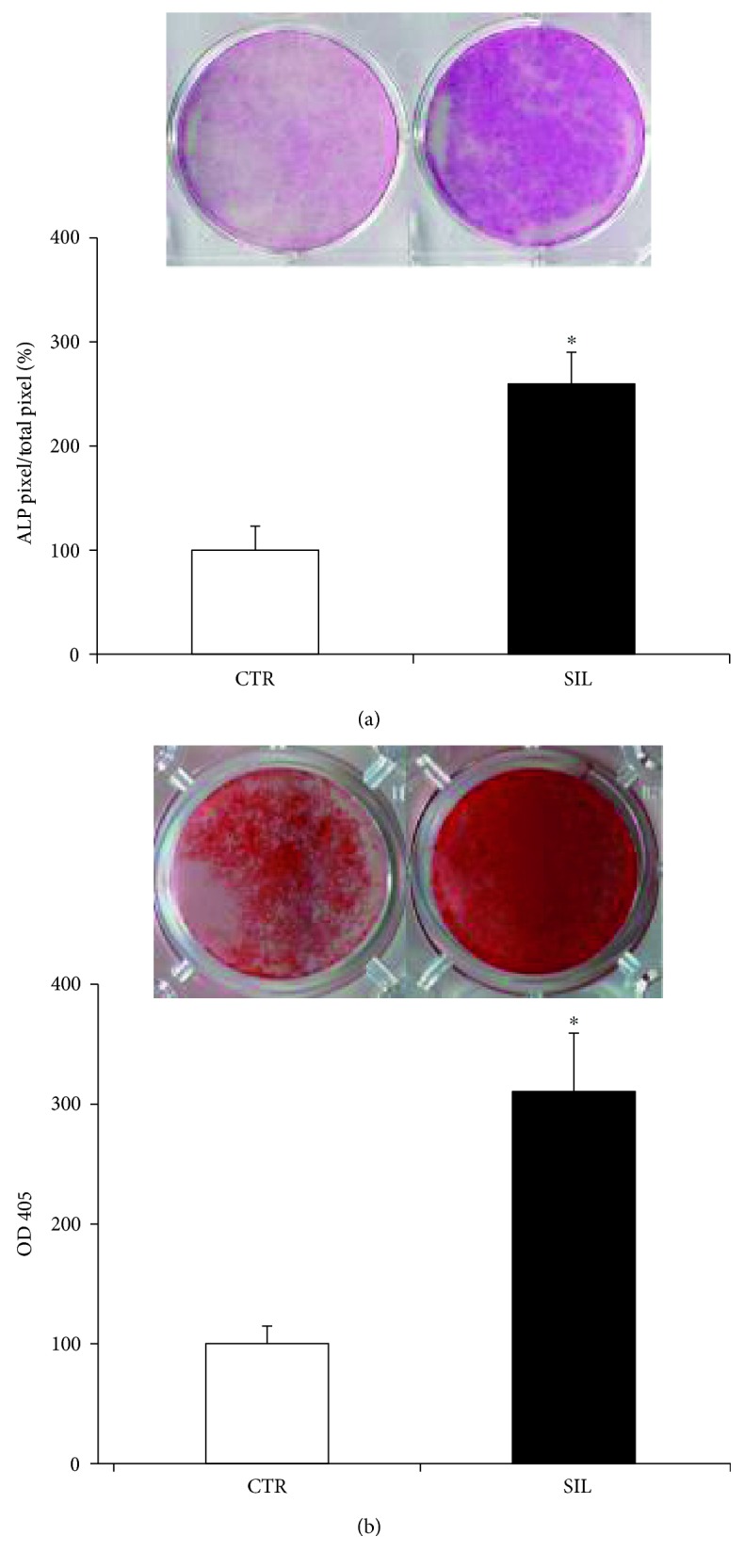
Effect of NURR1 downregulation on ALP and mineralization. (a) ALP histochemical assay (purple staining) performed on DPSCs transfected with NURR1-specific siRNA or scrambled sequences and maintained in osteogenic conditions for 7 days. The graph represents the quantification of positive staining as percentage compared to CTR (^∗^*P* < 0.01) and is representative for 3 independent donors. Data are presented as mean ± SEM. Student's 𝑡-test was used for single comparisons. (b) Mineral matrix deposition assayed by ARS (red staining) in siNURR1 and CTR cells after 21 days in osteogenic conditions. The graph shows the OD quantification of extracted dye from stained cell layers as percentage compared to CTR (^∗^*P* < 0.001) and is representative for 3 independent donors. Data are presented as mean ± SEM. Student's 𝑡-test was used for single comparisons.

However, the osteogenic markers that were significantly influenced by NURR1 downregulation have been described in details below. The expression of the typical osteoblast early markers Col1 and ALP was determined by qPCR (Figure 3). Col1 mRNA level increased in the CTR cells, along the analyzed differentiation steps (Figure 3(a)), as well as ALP (Figure 3(b)), confirming that DPSCs cultivated in osteogenic medium acquired the typical osteoblastic features. Intriguingly, the expression of Col1 significantly increased in NURR1 silenced cells compared to CTR cells with a significant trend at 6 and 8 days, as did ALP at 8 days, suggesting that NURR1 downregulation favors the osteogenic differentiation of DPSCs. The expression trend of Col1 was further confirmed, in NURR1 silenced and CTR cells, by Western blot analysis. As shown in Figure 3(c), Col1 protein level increased in NURR1 silenced cells if compared with CTR cells at 2–6 days, thus confirming the mRNA data. The molecular result of ALP trend was further supported by the histochemical evaluation of ALP expression. The histochemical assay was performed on DPSCs CTR and siNURR1 after 8 days of osteogenic differentiation (Figure 4(a)). As revealed by the purple staining, ALP expression was significantly more abundant in siNURR1 cells compared to CTR cells (~150%) corroborating the idea that NURR1 expression must be downregulated to prompt the cells to osteogenic lineage.

### 2.4. Downregulation of NURR1 in DPSCs Favors the Mineral Matrix Deposition Ability

To further investigate the role of NURR1 in osteoblast differentiation of DPSCs, we cultured CTR and NURR1 silenced cells in mineralizing conditions. The silencing sequences (siNURR1) or scramble (CTR) were added every 48 hrs in order to keep NURR1 downregulated. A histochemical assay was used to analyze how NURR1 knockdown could influence the ability of DPSCs to mineralize. As showed in Figure 4(b), the capacity of DPSCs to mineralize was highly enhanced in NURR1 silenced cells compared to CTR cells (200%). These results are in agreement with the increased expression of osteoblast markers Col1 and ALP and confirmed the finding that NURR1 is expressed in undifferentiated DPSCs, but down levels of the receptor prompt the differentiation of the cells toward the osteoblastic lineage and mineral matrix deposition.

## 3. Discussion

So far, NURR1 has been considered primarily involved in dopaminergic neurons differentiation and activity. Interestingly, a key role for the receptor was attributed during the differentiation of DPSCs toward a neuronal phenotype [19, 31–33]. Indeed, NURR1 is crucial for dopaminergic neuron function [37] and its malfunction has been correlated with neurological and inflammatory disease [38, 39]. By contrast, literature data about NURR1 role in osteoblasts are controversial: studies in mice highlighted an effect in increasing the osteoblastic phenotype of primary culture and osteoblastic cell lines [34, 35], while a more recent work indicated that NURR1 downregulated the main osteoblastic differentiation pathway, involving *β*-catenin, in a human osteoblastic cell line [36]. In addition, we found that MSCs such as DPSCs express NURR1 in basal and osteogenic conditions [23]. Thus, NURR1 is expressed in DPSCs, with a prominent role in neuronal differentiation, but its role in the osteogenic differentiation needs more investigations. Consistent with the essential role played by NURR1 in nervous tissue, we speculated if the molecule could, in some way, influence the DPSC differentiation toward the osteoblastic phenotype and, more precisely, if NURR1 inhibition could interfere with DPSC osteoblastogenesis. Primarily, we studied the neuronal marker nestin and the neuroglia marker G-Fap during DPSC osteogenic process. Both markers were expressed in DPSCs during the first phases of osteogenic differentiation, perhaps the cells still retaining a neuronal potency, but dramatically decreased after 8 days of culture, indicating the expected result that osteoblast differentiation triggers decreased DPSC neuronal potential. Tissue regeneration, based on adult stem cells approach, is still facing with strategies directed to control and increase their differentiation capacity; thus, the discovery of target molecules to modulate, in order to address the desired commitment, is still an open challenge [40]. Mainly, MSC multipotency has the problem in regeneration therapy to drive cell differentiation to the correct lineage, reconstructing the expected mature tissue. The role of NURR1 in osteoblast differentiation is not yet clearly established and is intriguing, since it is expressed in MSCs during osteoblastogenesis [23, 34, 36], but it is also involved in neuronal differentiation [19, 41]. To unambiguously establish the role of this receptor in osteogenic differentiation of MSCs, we inhibited NURR1 during all the processes submitting DPSCs to a repeated multistep silencing treatment. Primarily, we checked the successful of NURR1 silencing treatment at each time of the experiment. Hence, the main osteogenic markers were studied. Col1 expression indicated that DPSCs acquired the capacity to secrete the main bone matrix protein and we found that NURR1 silencing increased both mRNA and protein expression. ALP mRNA levels dramatically increased in silenced cells and the histochemical assay confirmed the different enzyme quantities, indicating that NURR1 downregulation had a strong effect on the expression of the molecule crucial for osteoblast during the matrix deposition. The final crucial step in the bone regenerative process is the inorganic matrix formation [42]. Thus, mature osteoblasts, after the secretion of organic matrix components, begin the mineralization phase. MSCs from dental tissues have been demonstrated to correctly undergo to mineralization process [43]: some substances such as vitamin D could increase the mineral matrix deposition [44]; we found that inhibiting NURR1 enhanced DPSC mineralization. In summary, NURR1 is expressed in DPSCs, but to pursuit the cells toward a greater matrix deposition, proper of mature osteoblast, the receptor can be downregulated. MSC differentiation fate can be artificially modulated, in vitro, by the appropriate culture conditions and compounds. Apparently, the epigenetic science indicates that different stimuli can interfere with gene expression; in vivo, this issue regards also cell differentiation. In conclusion, our results showed the expression of nestin and GFAP in DPSCs confirming their neural potential. In addition, we demonstrated that such neural and glial markers are still present during the first steps of osteogenic differentiation, suggesting that DPSCs still maintain quite a multipotency or perhaps not all the cells in the culture are yet committed to osteogenic lineage. After 8 days, the expression of these markers dramatically decreased, suggesting that the cells lose their neural potential. In the same way, we found that NURR1 is expressed in DPSCs, but keeping down its expression during the osteogenic differentiation, the expression of typical osteoblastic markers is increased, culminating in higher production of mineralized matrix. We demonstrated that one of the mechanisms regulating MSC plasticity, influencing their phenotype, is NURR1 expression; in particular, its inhibition promotes osteoblastogenesis and enhances mineral matrix deposition. Discovering an appropriate in vivo method for inhibiting NURR1 during MSC osteogenic differentiation could improve an adult stem cell based tissue engineering, enhancing bone tissue regeneration.

## 4. Patients, Materials, and Methods

### 4.1. Cell Cultures

Human pulp tissues were collected from the third molars of twenty healthy young adults aged between eighteen and twenty-six years. The study was approved by the Institutional Review Board of the Department of Dental Science and Surgery—Unit of Periodontology, University of Bari; the patients gave written informed consent. Once the teeth were extracted, the pulp tissues were dissected, enzymatically digested, and filtered to obtain single-cell suspensions. DPSCs harvested were seeded and expanded as previously described [2, 23, 45, 46]. For differentiation toward osteogenic lineage, cell culture medium was supplemented with 10^−8^ M dexamethasone and 50 *μ*g/ml ascorbic acid (Sigma Aldrich, Milan, Italy). For induction of matrix mineralization, we supplemented the cell culture medium with 10^−8^ M dexamethasone, 50 *μ*g/ml ascorbic acid, and 10 mM *β*-glycerophosphate.

### 4.2. Short-Interfering RNA Knockdown

DPSCs were transfected with NURR1-specific siRNA or scrambled sequences as control (50 nM) (Life Technologies) using RNAi Max Lipofectamine (Life Technologies). Both specific and control sequences were added on each medium change every 2 days, until the end of the culture, in order to keep the protein downregulated, reaching an optimal knockdown of NURR1 mRNA and protein (Figures 2(a) and 2(b)).

### 4.3. Real-Time RT-PCR

Total RNA was isolated using spin columns (RNasy, Qiagen, Hilden, Germany) according to the manufacturer's instructions and reverse transcribed (2 *μ*g) using the Superscript First-Strand Synthesis System kit (Invitrogen Life Technologies, Carlsbad, CA, USA); the resulting cDNA (20 ng) was subjected to quantitative PCR as described. Real-time PCR analysis of mRNA was performed using a BioRad CFX96 Real Time System using the SYBR green PCR method according to the manufacturer's instruction (BioRad iScript Reverse Transcription Supermix cat. 170-8841). The mean cycle threshold value (Ct) from triplicate samples was used to calculate gene expression, and PCR products were normalized to GAPDH levels for each reaction.

### 4.4. Immunoblotting

Total cell lysates were obtained as previously described [44, 45]. Total protein concentration was measured using the Bio-Rad Protein Assay kit, and cell lysates were separated by SDS-PAGE before transfer onto nitrocellulose membranes (Invitrogen, Carlsbad, CA). After immunoblotting with the appropriate antibodies, immune complexes were visualized by incubation with IRDye-labeled secondary antibodies (680/800CW) (LI-COR Biosciences, NE). For immunoblotting, the Odyssey infrared imaging system was used (LI-COR Corp., Lincoln, NE).

### 4.5. Alkaline Phosphatase (ALP)

The levels of the biochemical marker for the osteoblast activity, ALP, was tested in DPSC cultures differentiated with osteogenic factors, using the Leukocyte Alkaline Phosphatase Kit (Sigma Aldrich). Cells were fixed, gently washed with deionized water, and stained with ALP solution according to the manufacturer's instructions for 15′. After incubation, the cells were rinsed with water, air-dried, and then analyzed under the microscope. ALP-positive cells show a purple color. ALP quantification was performed by ImageJ, analyzing the number of colored pixels corresponding to the positive stained cells.

### 4.6. Alizarin Red Staining (ARS)

The capacity of differentiated DPSCs to produce calcium-rich deposits was analyzed by using alizarin red staining. The cells were gently rinsed with PBS, fixed with 10% formalin at room temperature for 10 minutes, and then rinsed again with deionized water. The staining was performed by adding 1% of ARS solution at room temperature for 10 minutes. After discarding the ARS solution, the wells were rinsed twice with deionized water and air-dried. Calcium-rich deposits appeared red stained. As previously described, the dye was extracted from the stained cell layer and assayed for quantification at 405 nm [46, 47]. Briefly, 10% acetic acid was added for 30 min at room temperature with shaking, the solution incubated 10 min at 85°C and then kept on wet ice for 5 min. Before reading the optical density at 405 nm, 10% ammonium hydroxide was added to neutralize the acid. The results were evaluated for statistical analysis.

## Figures and Tables

**Table 1:** Osteogenic markers in DPSC cultures: NURR1 silenced versus CTRs.

	2 days	4 days	6 days	8 days
ALP	UN	+	++	++
Col1	UN	++	++	++
Runx-2	+	UN	+	−
OPG	UN	−	−	++
OPN	+	−	−	UN
OCN	NF	NF	+	+

The table reports the osteogenic markers analyzed at 2, 4, 6, and 8 days of differentiation in NURR1 silenced and CTR cultures. The symbols indicate the ratio of NURR1 SIL compared to CTR cultures (expressed as %). + corresponds to an increase 10–50%; ++ corresponds to an increase higher than 50%; − corresponds to a decrease 10–50%; UN indicates a not significant variation; NF indicates a not relevant detection of the marker.

## References

[1] Gronthos S., Brahim J., Li W. (2002). Stem cell properties of human dental pulp stem cells. *Journal of Dental Research*.

[2] Gronthos S., Mankani M., Brahim J., Robey P. G., Shi S. (2000). Postnatal human dental pulp stem cells (DPSCs) in vitro and in vivo. *Proceedings of the National Academy of Sciences of the United States of America*.

[3] Sloan A. J., Smith A. J. (2007). Stem cells and the dental pulp: potential roles in dentine regeneration and repair. *Oral Diseases*.

[4] About I., Murray P. E., Franquin J. C., Remusat M., Smith A. J. (2001). The effect of cavity restoration variables on odontoblast cell numbers and dental repair. *Journal of Dentistry*.

[5] Murray P. E., About I., Franquin J. C., Remusat M., Smith A. J. (2001). Restorative pulpal and repair responses. *Journal of the American Dental Association (1939)*.

[6] Hanks C. T., Fang D., Sun Z., Edwards C. A., Butler W. T. (1998). Dentin-specific proteins in MDPC-23 cell line. *European Journal of Oral Sciences*.

[7] Ueno A., Kitase Y., Moriyama K., Inoue H. (2001). MC3T3-E1-conditioned medium-induced mineralization by clonal rat dental pulp cells. *Matrix Biology: Journal of the International Society for Matrix Biology*.

[8] Unda F. J., Martin A., Hilario E., Begue-Kirn C., Ruch J. V., Arechaga J. (2000). Dissection of the odontoblast differentiation process in vitro by a combination of FGF1, FGF2, and TGFbeta1. *Developmental Dynamics: An Official Publication of the American Association of Anatomists*.

[9] About I., Bottero M. J., Denato P., Camps J., Franquin J. C., Mitsiadis T. A. (2000). Human dentin production in vitro. *Experimental Cell Research*.

[10] Couble M. L., Farges J. C., Bleicher F., Perrat-Mabillon B., Boudeulle M., Magloire H. (2000). Odontoblast differentiation of human dental pulp cells in explant cultures. *Calcified Tissue International*.

[11] Giorgini E., Conti C., Ferraris P. (2011). FT-IR microscopic analysis on human dental pulp stem cells. *Vibrational Spectroscopy*.

[12] d'Aquino R., Rosa A., Laino G. (2009). Human dental pulp stem cells: from biology to clinical applications. *Journal of Experimental Zoology Part B, Molecular and Developmental Evolution*.

[13] Huang G. T., Gronthos S., Shi S. (2009). Mesenchymal stem cells derived from dental tissues vs. those from other sources: their biology and role in regenerative medicine. *Journal of Dental Research*.

[14] Nuti N., Corallo C., Chan B. M., Ferrari M., Gerami-Naini B. (2016). Multipotent differentiation of human dental pulp stem cells: a literature review. *Stem Cell Reviews*.

[15] Papaccio G., Graziano A., d'Aquino R. (2006). Long-term cryopreservation of dental pulp stem cells (SBP-DPSCs) and their differentiated osteoblasts: a cell source for tissue repair. *Journal of Cellular Physiology*.

[16] Spath L., Rotilio V., Alessandrini M. (2010). Explant-derived human dental pulp stem cells enhance differentiation and proliferation potentials. *Journal of Cellular and Molecular Medicine*.

[17] Tirino V., Paino F., Rosa A., Papaccio G. (2012). Identification, isolation, characterization, and banking of human dental pulp stem cells. *Methods in Molecular Biology (Clifton, New Jersey)*.

[18] Arthur A., Rychkov G., Shi S., Koblar S. A., Gronthos S. (2008). Adult human dental pulp stem cells differentiate toward functionally active neurons under appropriate environmental cues. *Stem Cells (Dayton, Ohio)*.

[19] Kanafi M., Majumdar D., Bhonde R., Gupta P., Datta I. (2014). Midbrain cues dictate differentiation of human dental pulp stem cells towards functional dopaminergic neurons. *Journal of Cellular Physiology*.

[20] Young F. I., Telezhkin V., Youde S. J. (2016). Clonal heterogeneity in the neuronal and glial differentiation of dental pulp stem/progenitor cells. *Stem Cells International*.

[21] Zhang J., Lian M., Cao P. (2016). Effects of nerve growth factor and basic fibroblast growth factor promote human dental pulp stem cells to neural differentiation. *Neurochemical Research*.

[22] Laino G., d'Aquino R., Graziano A. (2005). A new population of human adult dental pulp stem cells: a useful source of living autologous fibrous bone tissue (LAB). *Journal of Bone and Mineral Research: The Official Journal of the American Society for Bone and Mineral Research*.

[23] Mori G., Brunetti G., Oranger A. (2011). Dental pulp stem cells: osteogenic differentiation and gene expression. *Annals of the New York Academy of Sciences*.

[24] Mori G., Centonze M., Brunetti G. (2010). Osteogenic properties of human dental pulp stem cells. *Journal of Biological Regulators and Homeostatic Agents*.

[25] Menicanin D., Bartold P. M., Zannettino A. C., Gronthos S. (2010). Identification of a common gene expression signature associated with immature clonal mesenchymal cell populations derived from bone marrow and dental tissues. *Stem Cells and Development*.

[26] Shi S., Gronthos S. (2003). Perivascular niche of postnatal mesenchymal stem cells in human bone marrow and dental pulp. *Journal of Bone and Mineral Research: The Official Journal of the American Society for Bone and Mineral Research*.

[27] Shi S., Robey P. G., Gronthos S. (2001). Comparison of human dental pulp and bone marrow stromal stem cells by cDNA microarray analysis. *Bone*.

[28] Huang W., Yang S., Shao J., Li Y. P. (2007). Signaling and transcriptional regulation in osteoblast commitment and differentiation. *Frontiers in Bioscience: A Journal and Virtual Library*.

[29] Mori G., Brunetti G., Colucci S. (2007). Alteration of activity and survival of osteoblasts obtained from human periodontitis patients: role of TRAIL. *Journal of Biological Regulators and Homeostatic Agents*.

[30] Grand J. N., Gonzalez-Cano L., Pavlou M. A., Schwamborn J. C. (2015). Neural stem cells in Parkinson’s disease: a role for neurogenesis defects in onset and progression. *Cellular and Molecular Life Sciences: CMLS*.

[31] Jarmalaviciute A., Tunaitis V., Strainiene E. (2013). A new experimental model for neuronal and glial differentiation using stem cells derived from human exfoliated deciduous teeth. *Journal of Molecular Neuroscience: MN*.

[32] Majumdar D., Kanafi M., Bhonde R., Gupta P., Datta I. (2016). Differential neuronal plasticity of dental pulp stem cells from exfoliated deciduous and permanent teeth towards dopaminergic neurons. *Journal of Cellular Physiology*.

[33] Yang K. L., Chen M. F., Liao C. H., Pang C. Y., Lin P. Y. (2009). A simple and efficient method for generating Nurr1-positive neuronal stem cells from human wisdom teeth (tNSC) and the potential of tNSC for stroke therapy. *Cytotherapy*.

[34] Lee M. K., Choi H., Gil M., Nikodem V. M. (2006). Regulation of osteoblast differentiation by Nurr1 in MC3T3-E1 cell line and mouse calvarial osteoblasts. *Journal of Cellular Biochemistry*.

[35] Pirih F. Q., Tang A., Ozkurt I. C., Nervina J. M., Tetradis S. (2004). Nuclear orphan receptor Nurr1 directly transactivates the osteocalcin gene in osteoblasts. *The Journal of Biological Chemistry*.

[36] Rajalin A. M., Aarnisalo P. (2011). Cross-talk between NR4A orphan nuclear receptors and beta-catenin signaling pathway in osteoblasts. *Archives of Biochemistry and Biophysics*.

[37] Satoh J., Kuroda Y. (2002). The constitutive and inducible expression of Nurr1, a key regulator of dopaminergic neuronal differentiation, in human neural and non-neural cell lines. *Neuropathology: Official Journal of the Japanese Society of Neuropathology*.

[38] Altar C. A., Vawter M. P., Ginsberg S. D. (2009). Target identification for CNS diseases by transcriptional profiling. *Neuropsychopharmacology: Official Publication of the American College of Neuropsychopharmacology*.

[39] Vivekanantham S., Shah S., Dewji R., Dewji A., Khatri C., Ologunde R. (2015). Neuroinflammation in Parkinson’s disease: role in neurodegeneration and tissue repair. *The International Journal of Neuroscience*.

[40] Amini A. R., Laurencin C. T., Nukavarapu S. P. (2012). Bone tissue engineering: recent advances and challenges. *Critical Reviews in Biomedical Engineering*.

[41] Long X., Olszewski M., Huang W., Kletzel M. (2005). Neural cell differentiation in vitro from adult human bone marrow mesenchymal stem cells. *Stem Cells and Development*.

[42] Bonucci E. (2012). Bone mineralization. *Frontiers in Bioscience (Landmark Edition)*.

[43] Clarke B. (2008). Normal bone anatomy and physiology. *Clinical Journal of the American Society of Nephrology: CJASN*.

[44] Posa F., Benedetto A., Colaianni G. (2016). Vitamin D effects on osteoblastic differentiation of mesenchymal stem cells from dental tissues. *Stem Cells International*.

[45] Benedetto A., Brunetti G., Posa F. (2015). Osteogenic differentiation of mesenchymal stem cells from dental bud: role of integrins and cadherins. *Stem Cell Research*.

[46] Benedetto A., Carbone C., Mori G. (2014). Dental pulp stem cells isolation and osteogenic differentiation: a good promise for tissue engineering. *Methods in Molecular Biology (Clifton, New Jersey)*.

[47] Gregory C. A., Gunn W. G., Peister A., Prockop D. J. (2004). An Alizarin red-based assay of mineralization by adherent cells in culture: comparison with cetylpyridinium chloride extraction. *Analytical Biochemistry*.

